# Poxvirus-based vaccine therapy for patients with advanced pancreatic cancer

**DOI:** 10.1186/1479-5876-5-60

**Published:** 2007-11-26

**Authors:** Howard L Kaufman, Seunghee Kim-Schulze, Kelledy Manson, Gail DeRaffele, Josephine Mitcham, Kang Seok Seo, Dae Won Kim, John Marshall

**Affiliations:** 1 The Tumor Immunology Laboratory, Division of Surgical Oncology, Columbia University, New York, NY, USA; 2 The Lombardi Cancer Center, Georgetown University Medical Center, Washington, DC, USA

## Abstract

**Purpose:**

An open-label Phase 1 study of recombinant prime-boost poxviruses targeting CEA and MUC-1 in patients with advanced pancreatic cancer was conducted to determine safety, tolerability and obtain preliminary data on immune response and survival.

**Patients and methods:**

Ten patients with advanced pancreatic cancer were treated on a Phase I clinical trial. The vaccination regimen consisted of vaccinia virus expressing tumor antigens carcinoembryonic antigen (CEA) and mucin-1 (MUC-1) with three costimulatory molecules B7.1, ICAM-1 and LFA-3 (TRICOM) (PANVAC-V) and fowlpox virus expressing the same antigens and costimulatory molecules (PANVAC-F). Patients were primed with PANVAC-V followed by three booster vaccinations using PANVAC-F. Granulocyte-macrophage colony-stimulating factor (GM-CSF) was used as a local adjuvant after each vaccination and for 3 consecutive days thereafter. Monthly booster vaccinations for up to 12 months were provided for patients without progressive disease. Peripheral blood was collected before, during and after vaccinations for immune analysis.

**Results:**

The most common treatment-related adverse events were mild injection-site reactions. Antibody responses against vaccinia virus was observed in all 10 patients and antigen-specific T cell responses were observed in 5 out of 8 evaluable patients (62.5%). Median overall survival was 6.3 months and a significant increase in overall survival was noted in patients who generated anti CEA- and/or MUC-1-specific immune responses compared with those who did not (15.1 vs 3.9 months, respectively; *P *= .002).

**Conclusion:**

Poxvirus vaccination is safe, well tolerated, and capable of generating antigen-specific immune responses in patients with advanced pancreatic cancer.

## Introduction

Pancreatic cancer is associated with a poor prognosis and high mortality rate. An estimated 37,170 new cases will be diagnosed in the United States in 2007 and 33,370 patients will die from the disease [1]. The median overall survival rate in patients with unresectable or metastatic disease is typically 3–6 months [2]. Current treatment options for pancreatic cancer include surgery, chemotherapy, and radiotherapy but only complete surgical resection is associated with a favorable outcome [1,3]. Most patients with pancreatic cancer, however, have unresectable disease at presentation. Gemcitabine-based therapy is widely used in the treatment of advanced pancreatic cancer, although its benefits are primarily palliative with limited improvement in survival [4]. Thus, new therapeutic options are needed for patients with advanced pancreatic cancer.

There has been interest in using immunotherapy for pancreatic cancer based on the identification of mutated oncogenes, such as *KRAS*, altered tumor suppressor genes, such as *TP53*, *CDKN2A*, *DPC4*, *BRCA2*, and *ERBB2*, as well as over-expression of tumor-associated antigens, such as *CEA *and *MUC-1*, in pancreatic carcinoma cells [5]. The carcinoembryonic antigen (CEA) is an oncofetal antigen that is expressed at high levels in most pancreatic carcinomas. The mucin, MUC-1, is a highly glycosylated protein that is also overexpressed in many adenocarcinomas, including those of pancreatic origin. T cells from normal donors and cancer patients have been shown to recognize HLA-restricted epitopes derived from CEA and non-HLA-restricted epitopes encoded by MUC-1 [6,7]. Targeting two distinct tumor antigens in a single vaccination regimen may induce better anti-tumor effects since the generation of polyclonal T cell responses may prevent tumor escape through antigen loss. Several preclinical models have demonstrated that the T cell dependent therapeutic effectiveness of using recombinant poxviruses expressing CEA or MUC-1 in both transplantable and transgenic model tumor systems [8-17].

The activation of T cells requires antigen-specific signals that are delivered through the T cell receptor after recognizing cognate peptides presented by major histocompatibility complexes (MHC) on antigen-presenting cells. In the face of weak antigenic stimuli, such as tumor antigens, successful activation of T cells also depends on costimulatory signals, which cooperate with T cell signaling to induce cytokine production and T-cell proliferation [17]. The co-expression of tumor antigens and costimulatory molecules within poxviruses represents a strategy that has demonstrated a significantly better anti-tumor effect in murine models [16,18-25]. The combination of B7.1, ICAM-1 and LFA-3 (TRICOM) appears to be particularly useful for both in vitro stimulation of T cells and for induction of tumor rejection in vivo [18,19,24,26].

In addition to expression of costimulatory molecules in poxvirus vectors, the use of a heterologous prime-boost vaccination schedule with replicating and non-replicating vectors has shown superiority in generating tumor-specific T cell responses in animal models [14]. Furthermore, pre- and clinical studies have shown that protection from malaria was enhanced when using a prime-boost vaccinia-fowlpox virus system expressing the *P. falciparum *circumsporozoite antigen (PfCSF) [27,28]. We have previously shown that prime-boost vaccination with a prostate specific antigen (PSA) vaccinia-fowlpox virus regimen induced a longer biochemical progression-free survival than vaccination with fowlpox-PSA virus alone [22]. In this study we tested the safety and feasibility of using poxviruses expressing CEA and MUC-1 with TRICOM as a vaccine treatment for patients with advanced pancreatic carcinoma. A novel vaccinia virus vectors expressing both tumor antigens CEA and MUC-1, and costimulatory molecules TRICOM (PANVAC-V) was constructed and used to prime an initial T-cell response. Patients were then boosted with a non-replicating fowlpox virus expressing CEA, MUC-1 and TRICOM (PANVAC-F). The use of a prime-boost vaccination approach with vaccinia virus followed by fowlpox virus has been shown to increase the generation of T cell immunity to expressed antigens and may also improve therapeutic responses [14,22,29,30]. In order to further improve the clinical effectiveness of the vaccine, granulocyte-macrophage colony-stimulating factor (GM-CSF) was used as a vaccine adjuvant to enhance local antigen processing and presentation [31,32]. Thus, this trial incorporated four distinct strategies to increase the potency of anti-tumor immunity in pancreatic cancer patients – targeting multiple tumor antigens, expression of several T-cell costimulatory molecules, delivery using a heterologous prime-boost poxvirus system and inclusion of GM-CSF as a local adjuvant. We now report the safety and tolerability of this approach as well as immune responses and their correlation to overall patient survival.

## Patients and methods

### Patient eligibility

The study populations comprised 10 patients ≥18 years old who had been previously vaccinated against smallpox and had a histologically confirmed diagnosis of unresectable or metastatic pancreatic cancer; Karnofsky Performance Status (KPS) ≥80%; and anticipated survival of ≥4 months. Patients were required to use adequate contraceptives during the study and for 3 months after the final visit. Key exclusion criteria included: evidence of being immunocompromised; past or present diagnosis of autoimmune disease; steroid use within 28 days prior to signing consent; inability to avoid close contact with children ≤5 years old, pregnant women, individuals with eczema or related skin conditions, and/or immunocompromised individuals for 3 weeks after the first vaccination; known egg or egg product allergy; positive for hepatitis B or C infection; compromised hematopoietic function; hepatic or renal dysfunction; significant cardiovascular abnormalities or other uncontrolled diseases or conditions; concurrent other malignancy, except non-melanoma skin or in situ cervix carcinoma; prior malignancy that had not been curatively treated or that had recurred within 2 years; evidence of active, uncontrolled infection; completion of prior chemotherapy <28 days prior to the first vaccination; receipt of immunotherapy or biotherapy; and pregnancy or breastfeeding.

The study was approved by the individual Institutional Review Boards at each study site and was conducted in accordance with the Declaration of Helsinki. Written informed consent was obtained from all patients prior to study entry. An independent Data Safety Monitoring Board was established to ensure patient safety.

### Study design and treatment

On Day 0, all patients were primed with rV-CEA/MUC-1/TRICOM (PANVAC-V, 2 × 10^8 ^pfu). This was followed by booster doses of rF-CEA/MUC-1/TRICOM (PANVAC-F, 1 × 10^9 ^pfu) on Days 14, 28, and 42. Each vaccine was administered by subcutaneous injection. In addition, GM-CSF 100 μg was administered at the vaccination site after each immunization and for 3 consecutive days thereafter. Patients were eligible to enroll in an optional extension phase provided they completed the core phase of 4 vaccinations and experienced disease stabilization with acceptable toxicity. For those patients enrolled in the extension phase, additional monthly boosts in combination with GM-CSF were provided for up to 12 months.

Adverse events were monitored throughout the trial by physical examinations, laboratory tests (chemistries and electrolytes, blood counts, coagulation parameters, hepatic enzymes and urinalysis), and vital sign measurements conducted on Days 0, 14, 28, 42, and 70 in the core phase, and at each monthly boost visit in the extension phase. Twelve-lead ECGs were performed on Days 28 and 70 in the core phase, and at monthly boost visits from months 3 through 11 in the extension phase. Peripheral blood was collected for immune studies on Days 0, 14, 28, 42, and 70 in the core phase, and at each monthly boost visit in the extension phase.

A patient was withdrawn from the study if any of the following dose-limiting toxicities (DLT) were experienced: grade 2 asymptomatic bronchospasm or generalized urticaria or any other grade ≥3 allergic reaction; grade ≥2 autoimmune response; or any grade ≥3 hematologic or nonhematologic reaction, including injection-site reaction.

### Vaccine preparation

PANVAC-V and PANVAC-F vaccines were prepared from plaque-purified isolates from the Wyeth New York City Board of Health (New York, NY) for vaccinia vectors and from POXVAC-TC for fowlpox vectors. Virus for the vaccine was grown in primary chicken embryo dermal cells and formulated in phosphate-buffered saline containing 10% glycerol. Each vial of PANVAC-V contained 1.29 × 10^9 ^pfu/0.3 mL, and PANVAC-F contained 8.93 × 10^9 ^pfu/0.3 mL. All vials were stored at -70°C or lower until the day of administration, at which time they were thawed at room temperature and prepared in a sterile manner.

### Evaluation of antibody responses

Serum was collected as mentioned above, stored at -20°C and used to determine anti-vaccinia virus, anti-fowlpox virus and anti-CEA antibody titers by standard ELISA, as previously described [33]. Briefly, 96-well plates were coated with the appropriate coating antigens overnight at 4°C. Wells were blocked for 1 hr at 37°C with 5% milk solution. Serum samples were diluted in 5% milk solution, with the starting sample at a 1:100 dilution and subsequent samples subjected to two-fold serial dilutions. The diluted test samples and assay positive and negative controls were added to the plates in duplicates. After incubation and washing, HRP-goat anti-human IgG was added and incubated for 1 hr at 37°C. Color was developed with Sure Blue TMB Microwell Peroxidase Substrate and absorbance was measured using a SpectraMax plate reader (Molecular Devices, CA). Antibody titers were defined as the mean optical density (O.D) of the test sera was ≥3 fold the mean O.D of the negative control at 1:100 dilution. A positive antibody response due to vaccination was defined as a ≥2 fold increase in the post-immunization titer as compared to the pre-immunization titer.

### Evaluation of T cell responses

PBMCs were separated from whole blood over a Ficoll gradient, and isolated PBMCs were frozen in liquid nitrogen until analysis. T cell responses were evaluated using a novel, research-grade, non-HLA-restricted cytokine secretion assay in all patients [34]. This assay compared the amount of IFN-γ produced in response to monkey breast cancer cells (CMMT 110/C1) infected with PANVAC-F with that produced in response to cells infected with negative control viruses, TBC-F/TRICOM™ or PROSTVAC-F. Briefly, frozen PBMC were thawed and incubated in medium overnight at 37°C, 5% CO_2 _prior to use. A CMMT 110/C1 cell line was infected with one of three different recombinant fowlpox viruses at a multiplicity of infection (MOI) of 40 pfu/cell: PANVAC-F, TBC-F/TRICOM (control fowlpox virus expressing TRICOM without CEA or MUC-1; data not shown) and PROSTVAC-F (control vector expressing PSA and TRICOM). On Day 2, each different infected CMMT 110/C1 preparation was mixed with patient PBMC at a ratio of 1:10 and co-cultured for 72 hours. At the end of the incubation period, supernatants were collected for IFN-γ production by standard ELISA assay using ELISA kit (R & D Systems). The limit of quantitation of IFN-γ in the ELISA was 50 pg/mL. When the number of viable patient PBMC permitted, the PBMC were also co-cultured with uninfected stimulator cells as an additional control. Under these conditions, IFN-γ production was not observed (data not shown).

### Statistical methods

Mean antibody titers against viral vectors, CEA and MUC-1 were determined for all evaluable patients by ELISA. The development of a specific cell-mediated immune response to CEA and MUC-1 was determined by comparing the amount of IFN-γ produced in response to monkey cells infected with PANVAC-F to the amount produced in response to cells infected with the negative control viruses, TBC-F/TRICOM or PROSTVAC-F. The Spearman correlation was used to investigate potential associations between response and clinical benefit. A *P*-value < 0.05 was considered significant.

## Results

### Patient characteristics

Eleven patients were enrolled in the trial since one patient withdrew consent before completing the core phase and was replaced. All patients were evaluated for toxicity but only the 10 patients completing the study were use for immune analysis. Patient demographics and baseline characteristics are listed in Table 1. The majority of patients were Caucasian, with a mean age of approximately 57.9 ± 9.6 (43–74) years. All patients were heavily pretreated with 60% had two or more prior chemotherapy regimens. Over half of the patients had undergone surgical procedures for pancreatic cancer and the majority (80%) had metastatic disease.

**Table 1:** Patient demographics

**Characteristics**	**N = 10**
Mean (range) age, years	57.9 ± 9.6 (43–74)
Performance status*, n (%)	
80%	4 (40)
90%	3 (30)
100%	3 (30)
Sex, n (%)	
Male	7 (70)
Female	3 (30)
Race, n (%)	
Caucasian	9 (90)
Blacks/Asians	1 (10)
Prior therapy, n (%)	
Chemotherapy	
1 prior regimen	4 (40)
≥2 prior regimens	6 (60)
Radiotherapy	5 (50)
Immunotherapy	0 (0)
Other	1 (10)
HLA-A2†, n (%)	
Positive	3 (30)
Negative	7 (70)

Abbreviation: †HLA-A2, human leukocyte antigen-A2; * Karnofsky Performance Status (KPS)

### Safety

Toxicity was assessed using the National Cancer Institute Common Toxicity Criteria. The majority of adverse events were low grade injection site reactions or constitutional symptoms (Table 2). The most frequently encountered adverse events were grade 1 discomfort at the injection site (pain, erythema, edema; *n *= 5), constitutional symptoms (fatigue, mylagias, headache; *n *= 9), and gastrointestinal (nausea, vomiting, anorexia; *n *= 12). There were no Grade 3 or greater adverse events related to vaccination. No patients in the trial discontinued because of an adverse event.

**Table 2:** Adverse events related to vaccination

**Toxicity**	**Total Events (N = 10)**	**Grade 1**	**Grade 2**	**Grade 3**
***Injection site reactions***	5	5	0	0
***Fever or chills***	2	1	1	0
***Fatigue***	6	3	3	0
***Myalgia***	1	1	0	0
***Headache***	2	0	2	0
***Nausea***	4	2	2	0
***Vomiting***	3	1	2	0
***Anorexia***	5	5	0	0

### Antibody responses

Anti-poxvirus- and CEA-specific antibody responses were monitored by ELISA at each sampling time point throughout the trial and expressed as a titer (Table 3). All 10 patients developed antibody responses against vaccinia virus with 3 patients exhibiting titers >1000, although all patients had received prior smallpox vaccine. Seven of 10 patients (70%) developed a significant increase in anti-fowlpox virus titers by day 70 with one patient exhibiting a 16-fold increase 42 days after completing the assigned treatment. Similar to previous CEA-based vaccine trials, the anti-CEA antibody titers were much lower than anti-fowlpox virus titers [15,35]. Nonetheless, we detected an increase in anti-CEA antibody titers in 5 of 10 (50%) patients following vaccination with one patient demonstrating persistent anti-CEA antibody titers up to 3 months after completing the core phase of the trial.

**Table 3:** Antibody responses to vaccine, fowlpox and CEA

**Patient No.**	**Time point**	**Titer**		
		
		**Anti-Vaccinia**	**Anti-Fowlpox**	**Anti-CEA**
002-001	Day 0	1600	200	ND^a^
	Day 14	**6400**	-	<100
	Day 28	-	-	<100
	Day 42	-	-	<100
	Day 70	-	**3200**	<100
	Extension Phase Month 1^b^	-	-	<100
	Extension Phase Month 2	-	-	<100
002-002	Day 0	1600	<100	ND
	Day 14	**3200**	-	<100
	Day 28	-	-	<100
	Day 42	-	-	<100
	Day 63	-	**1600**	<100
002–003	Day 0	400	<100	ND
	Day 14	**1600**	-	<100
	Day 28	-	<100	<100
	Day 36	-	-	ND
002–005	Day 0	200	<100	ND
	Day 14	**800**	-	<100
	Day 28	-	-	<100
	Day 42	-	-	100
	Day 70	-	**400**	<100
002–006	Day 0	200	<100	ND
	Day 14	**800**	-	<100
	Day 28	-	-	<100
	Day 42	-	-	**3200**
	Day 70	-	**800**	**800**
	Extension Phase Month 2	-	-	**800**
	Extension Phase Month 3	-	-	**400**
002–007	Day 0	<100	200	ND
	Day 14	200	-	<100
	Day 28	-	-	<100
	Day 42	-	-	100
	Day 70	-	**800**	100
	Extension Phase Month 2	-	-	<100
	Extension Phase Month 3	-	-	<100
	Extension Phase Month 4	-	-	<100
	Extension Phase Month 5	-	-	<100
	Extension Phase Month 6	-	-	<100
	Extension Phase Month 7	-	-	<100
	Extension Phase Month 8	-	-	<100
	Extension Phase Month 9	-	-	<100
001–008	Day 0	800	<100	ND
	Day 14	**6400**	-	<100
	Day 28	-	-	<100
	Day 42	-	**3200**	<100
001–009	Day 0	800	<100	ND
	Day 14	800	-	<100
	Day 28	-	<100	<100
001–010	Day 0	400	<100	ND
	Day 14	**1600**	-	<100
	Day 28	-	-	<100
	Day 42	-	100	100
001–011	Day 0	1600	100	ND
	Day 14	1600	-	<100
	Day 28	-	-	<100
	Day 42	-	-	<100
	Day 70	-	**800**	200
	Extension Phase Month 2	-	-	100

Bolded values indicate development of antibody response, which is defined as a 2-fold increase in titer of the post-immunization sample as compared to the corresponding negative control sample. ^a^ND: not determined. Because the time 0 sample is the negative control sample for the CEA ELISA, a titer cannot be assigned to this sample. ^b^For this patient, the sample collected approximately 2 weeks after the day 70 vaccination was labeled "Extension Phase Month 1"; for all other patients, the sample collected one month after the day 70 vaccination was labeled "Extension Phase Month 2".

### T cell responses

**Figure 1:**
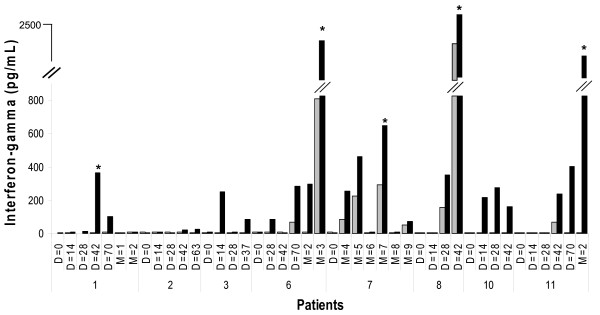
Cellular immune responses in vaccinated patients. Peripheral blood mononuclear cells were collected at the indicated day (D) or month (M) following initial immunization. Peripheral blood mononuclear cells were co-cultured with CMMT 110/C1 cells infected with a multiplicity of infection (MOI) of 40 plaque-forming units (pfu)/cell of fowlpox virus expressing CEA, MUC-1 and TRICOM (Black), or fowlpox virus expressing PSA and TRICOM; (Grey). *Denotes two-fold increase of CEA/MUC-1-specific T cell response compared to PSA control.

Antigen-specific immune responses were analyzed in eight patients using thawed PBMCs directly without any additional *in vitro *re-stimulation using a non-HLA-restricted cytokine secretion assay. In this assay autologous T cells were co-cultured with CMMT 110/C1 cells infected with PANVAC-F or a PSA-TRICOM expressing fowlpox virus, PROSTVAC-F to determine responses to the CEA and MUC-1 antigens in the PANVAC-F vaccine. Among the 8 patients with enough cells for evaluation, five patients (62.5%) developed a significant increase in antigen-specific (CEA or MUC-1) immune responses, which became detectable within 1–2 months of the first vaccination and generally increased with repeated boosting (Fig. 1). While no patients had detectable PANVAC-F specific T cell responses prior to PANVAC-F immunization, following prime-boost vaccination, positive responses were detected in 5 patients following PANVAC-F boosting. Two additional patients showed an initial induction of PANVAC-F specific T cells but the responses were not seen at later time points; these two patients were not deemed to have statistically positive cellular responses. The tumor antigen-specific nature of the T cell responses was suggested by the increased recognition of PANVAC-F compared to PROSTVAC-F, although two patients did react to a lesser degree to PROSTVAC-F (Fig. 1) likely representing recognition of fowlpox virus antigens.

### Immune response and overall survival

**Figure 2:**
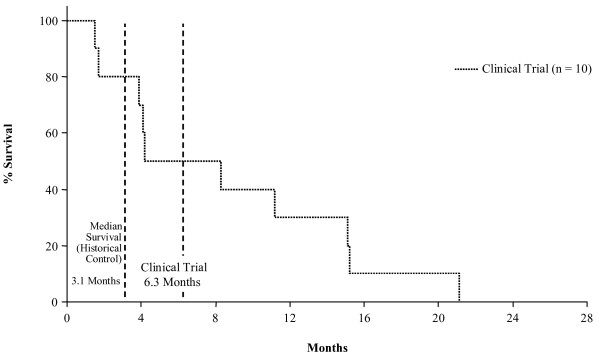
Overall survival of patients enrolled in trial compared with historical control. Median overall survival = 6.30 months (Trial), and 3.1 months (control).

**Figure 3:**
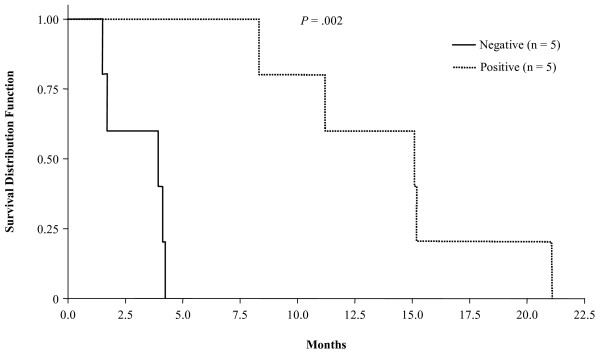
Overall survival of patients enrolled in the study. Positive indicates patients who had evidence of the development of a carcinoembryonic antigen (CEA)- and/or mucin-1 (MUC-1)-specific immune response. Negative indicates patients who did not develop an immune response (n = 3) or who were not assayed (n = 2).

The median overall survival was 6.3 months (range, 1.5–21.1 months; Fig. 2). Of note was the 1 year survival rate of 30%, although this trial was not designed to detect clinical responses. To explore whether there was an association between T cell response and clinical outcome we compared the overall survival of the five patients who demonstrated an increase in T cell response to the five who did not (Fig. 3). While these results must be interpreted cautiously, we did observe a significant increase in overall survival in the patients who generated vaccine-specific T cell responses compared to those without T cell reactivity (median survival 15.1 vs. 3.9 months, respectively; *P *= .002).

## Discussion

This Phase 1 clinical trial provides evidence that prime-boost vaccination with recombinant vaccinia and fowlpox viral vectors expressing CEA, MUC-1 and the TRICOM costimulatory molecules administered with local GM-CSF is safe and well tolerated. The most common treatment-related adverse events were mild injection-site reactions and no patients discontinued vaccination due to treatment-related adverse events. The vaccination regimen was also effective in generating antibody responses against the viral vectors and five patients generated an increase in anti-CEA antibody titers following vaccination. In agreement with other CEA-based vaccine trials we noted a relatively low titer of anti-CEA antibodies which may be related to the low immunogenicity of CEA or the presence of circulating CEA antigen-antibody complexes [33]. Nonetheless, even after subtracting background activity, we could still detect an increase in titers in five patients with one showing a dramatic increase at day 42, which remained elevated for at least 3 months. All patients developed anti-vaccinia antibody titers and some patients had pre-existing titers presumably related to prior smallpox vaccination, since immunity is known to persist for decades, in some cases [35]. Whether these high titers interfere with subsequent induction of T cell responses cannot be determined from our study due to the limited number of patients. In contrast, antibody titers against fowlpox virus were lower and this is consistent with the non-replicative nature of this virus.

We utilized a novel cytokine release assay using recombinant virus-infected CMMT 110/C1 cells as targets. This assay has several advantages for monitoring viral vaccine trials. The CMMT 110/C1 cell line represents a potentially useful alternative APC for antigen-presentation when autologous DC are limited or when multiple antigens need to be tested. These cells are highly permissive to poxvirus infection and human T cells are highly cross-reactive with rhesus monkey cells. This is due, in part to the evolutionary conservation of MHC-DR/peptide/T cell interactions between humans and rhesus monkeys [36,37]. Furthermore, Geluk et al. demonstrated that human T cells could proliferate in response to human hsp65_p3-13 _peptide when presented by rhesus APC *Mhc*-DRB1*03 [38]. In an experimental autoimmune encephalitis (EAE) model, human whole myelin basic protein (MBP) or purified MBP induced pathologic CNS lesions in rhesus monkeys presumably mediated by rhesus CD4^+ ^T cells [39]. These studies also suggested that the amplitude of the T cell response was comparable to that induced by human APC and confirmed that rhesus APC can efficiently process human antigens and provide co-stimulatory signals to human T cells. The permissiveness of the CMMT 110/C1 cell to poxvirus infection allows evaluation of T cell response against a full range of putative antigenic epitopes encoded by tumor antigens within the recombinant fowlpox virus vector.

In the current trial, we observed 5 of 8 (62.5%) patients in the study developed evidence of increased antigen-specific T cells within 2 months of vaccination and these persisted during the booster phase of the clinical trial. Although two patients also developed detectable levels of IFN-γ (>200 pg/ml) at one or more time points, they were not considered to have developed a significant responses to PANVAC-VF based on our validation cutoff levels. Nonetheless, this level of T cell response compares favorably with other trials of CEA and MUC-1-based vaccines [15,40]. Patients also developed T cell responses against fowlpox virus, although at much lower frequencies (Fig. 1). The data in our trial represents a small sample size, but these encouraging results suggest that our assay may be promising for monitoring other studies using viral vaccines.

Patients with pancreatic cancer may exhibit signs of active systemic immunosuppression due to prior chemotherapy and radiotherapy. Several studies have observed that cytotoxic CD8^+ ^T cells do not reach the tumor microenvironment in significant numbers because most of the cells aggregate in peritumoral tissues distant from the tumor cells [41,42]. The inactivation of T cells due to down-regulation of the adhesion molecule ligand (CD103^+^) and overexpression of immunosuppressive cytokines (TGF-β) observed in pancreatic cancer cells provide further evidence of immunosuppression in this population [43-45]. In addition, many patients with pancreatic cancer are heavily pretreated with chemotherapy, which may contribute to the highly suppressive nature of this patient population. In fact, all of our patients had been pre-treated with the majority having two or more prior chemotherapy regimens. Thus, the pancreatic cancer patient with advanced disease may represent a particularly difficult population to target with vaccines. Nonetheless, the detection of CEA-specific T cells in 62.5% of the vaccinated patients in this study suggest that vaccines would be more effective in induction of immunity in less immune suppressive environment as in patients with less advanced disease.

We also utilized local GM-CSF as a vaccine adjuvant in this trial, which is thought to promote local dendritic cell accumulation and presentation of virally-expressed antigens. This may have also played a role in improving the level of vaccine-specific immunity observe in our trial as other studies of irradiated GM-CSF-secreting allogeneic pancreatic tumor cell vaccines have also shown potent immune responses and potential therapeutic activity in early phase clinical trials [46]. Previous studies in patients with pancreatic cancer suggested that survival was closely correlated with the density of CD8^+ ^T cells within the tumor microenvironment [47]. More recently clinical investigation with poxviruses has similarly suggested an association between the generation of CEA-specific immunity and survival. Marshall and colleagues observed a similar effect and demonstrated increased progression-free survival in patients with CEA-expressing tumors who developed CEA-specific T cell responses following vaccination with recombinant poxviruses expressing CEA and costimulatory molecules [48]. The preliminary data from our Phase 1 clinical trial found a similar survival benefit in patients who developed CEA-specific T cell immunity and survival (see Figure 3). The median overall survival was nearly 4-fold longer in patients who exhibited evidence of an increased CEA-specific T cell response.

The Phase 1 clinical trial reported here establishes the preliminary safety and efficacy profiles of targeted cancer immunotherapy using a prime-boost vaccinia/fowl poxvirus vaccine regimen in patients with advanced pancreatic cancer. All patients developed anti-viral antibody titers and five patients developed anti-CEA antibody titers after vaccination. We also observed an increase in tumor antigen-specific T cell responses in 62.5% of the patients using a modified IFN-γ release assay with another two patients showing a non-significant increase. Importantly, we also documented an association between these T cell responses and overall survival, although this trial was not designed to detect such responses and therefore these results should be viewed as exploratory. In conclusion, vaccination with recombinant poxviruses generates meaningful antigen-specific immune responses even in heavily pre-treated pancreatic cancer patients. Future studies need to evaluate vaccine therapy in patients with less advanced disease, in those with less prior exposure to chemotherapy, and in combination with therapeutic strategies aimed at blocking immunosuppressive mechanisms. Such studies should help define the role of vaccine therapy for patients with pancreatic cancer.

## Authors' contributions

HLK conducted the clinical trial and contributed to the data analysis and manuscript preparation. SK-S contributed to the immunological analysis and manuscript preparation. KM conducted the immune assays. GD contributed to the screening and treatment of clinical study subjects. JM contributed to the data collection, entry and regulatory management of the clinical trial. KS contributed to the data analysis and manuscript preparation. DK contributed to the immune assay analysis and data interpretation. JM conducted the clinical trial at his institution and contributed to data analysis and manuscript preparation. All authors read and approved the final manuscript.
